# The Use of HSQoL-24 in an Assessment of Quality-of-Life Impairment among Hidradenitis Suppurativa Patients: First Look at Real-Life Data

**DOI:** 10.3390/jcm10225446

**Published:** 2021-11-22

**Authors:** Piotr K. Krajewski, Servando E. Marrón, Manuel Gomez-Barrera, Lucía Tomas-Aragones, Yolanda Gilaberte-Calzada, Jacek C. Szepietowski

**Affiliations:** 1 Department of Dermatology, Venereology and Allergology, Wroclaw Medical University, Chalubinskiego Street 1, 50-368 Wrocław, Poland; piotr.krajewski@student.umw.edu.pl; 2 Aragon Psychodermatology Research Group (GAI + PD), Dermatology Department, University Hospital Miguel Servet, 50009 Zaragoza, Spain; semarron@aedv.es (S.E.M.); ygilaberte@gmail.com (Y.G.-C.); 3 Department of Medicine, Psychiatry and Dermatology, University of Zaragoza, Paseo de Isabel la Catolica 1-3, 50009 Zaragoza, Spain; 4 Pharmacoeconomics & Outcomes Research Iberia (PORIB), Campus Universitario, University of San Jorge, Autovia Mudejar Km. 299, Villanueva de Gallego, 50830 Zaragoza, Spain; mgomezbarrera@gmail.com; 5 Department of Psychology, University of Zaragoza, Calle Pedro Cerbuna 12, 50009 Zaragoza, Spain; luciatomas@cop.es

**Keywords:** hidradenitis suppurativa, quality of life, HSQoL-24, burden

## Abstract

Hidradenitis suppurativa (HS) is a chronic inflammatory skin disorder with well-documented effects on patients’ quality of life (QoL). The aim of this study was to evaluate the QoL of patients with HS via the use of a newly developed questionnaire: Hidradenitis Suppurativa Quality of Life-24 (HSQoL-24). This study was performed on a population of 342 HS patients. Their QoL was assessed via the HSQoL-24 questionnaire. The perceived impairment of QoL due to HS in the studied group was considered to be serious (mean HSQoL-24 score: 58.3 ± 21.0 points). Women tended to experience a significantly higher impact from the disease than men (61.6 ± 19.2 points vs. 51.1 ± 23.1 points, *p* < 0.001). The HS severity had an effect on the perceived QoL, with statistically significant differences being evident between the self-assessed HS severity groups. The level of QoL impairment correlated positively with the number of affected body areas (*r* = 0.285, *p* < 0.001) and the duration of the disease (*r* = 0.173, *p* = 0.001), while the patients’ age at disease onset correlated negatively with the HSQoL-24 global score (*r* = −0.182, *p* = 0.001). Patients living in their family house scored higher than other groups. The least affected were patients who lived alone. The study shows that the HSQoL-24 questionnaire is a reliable, HS-specific tool for measuring the QoL among patients with HS in real-life clinical settings.

## 1. Introduction

In the last few decades, there has been a significant shift in the focus of medicine and research results towards patient-reported outcomes (PROs). The concept of disease-related quality of life (QoL) has been gradually integrated into both clinical and research practices [1]. This has changed the old medicine model, which concentrated on objective measures (like laboratory results and blood pressure), into a new, holistic model that concentrates on the patient and highlights the importance of their QoL. Nowadays, disease-related QoL is an important end point of many studies and clinical trials [2].

Since 1994, when Finlay and Khan [3] developed the Dermatology Life Quality Index (DLQI), multiple dermatology- and disease-specific QoL questionnaires have been created [4]. The authors of this paper were determined to create a good instrument that would properly reflect the level of QoL impairment in patients suffering from hidradenitis suppurativa (HS). HS is a chronic inflammatory condition that primarily affects apocrine-gland-rich regions of the body such as the axillary and groin areas. HS presents with painful nodules and abscesses that may coalesce and form fistulas where the pus has drained away. The lesions often evolve into scars that have a high physical and psychological impact on patients [5]. The above-mentioned symptoms, along with the purulent discharge and foul smell caused by the disease, make HS the most burdensome chronic dermatosis [6]. The lack of HS-specific questions in the majority of dermatology-specific QoL instruments makes them inadequate for the evaluation of QoL impairment in HS patients. Newer methods of measuring the PROs of HS patients, although they have been developed, still do not fully reflect the physical aspects of the disease [7]. Recently, a new, promising HS-specific instrument called Hidradenitis Suppurativa Quality of Life-24 (HSQoL24) was developed, validated, and translated into English by Marrón et al. [8,9].

The aim of this study was to determine the level of QoL impairment with the use of HSQoL24 among a large cohort of HS patients in a real-life setting.

## 2. Materials and Methods

### 2.1. Study Group

The study was performed on Spanish HS patients who were treated at the following hospitals in Spain between December 2018 and December 2020: the Miguel Servet University Hospital (Zaragoza), the Royo Villanova Hospital (Zaragoza), and the Barbastro Hospital (Huesca). All the patients were examined, diagnosed, and evaluated by a trained specialist in dermatology. The severity of the HS (assessed via the Hurley system and self-assessment), the duration of the disease, as well as the number of affected areas were recorded. Routine demographic data included gender, age, weight, height, education, marital status, and living situation. In line with the guidelines for studies involving human subjects and the World Medical Association Declaration of Helsinki, the anonymized data were then transferred to the Miguel Servet University Hospital of Zaragoza for a scientific evaluation. The study was accepted by the Ethics Committee of the University of Zaragoza (number PI16/020).

The studied group consisted of 342 consecutive HS patients (234 females and 108 males). The patients were 37.5 ± 10.7 years old. The mean BMI of the study participants was 29.3 ± 6.1 kg/m^2^, qualifying the population as overweight. The majority of the patients were living with their families (55.3%), had reached higher education (43.9%), and were professionally active (52%) (detailed demographic data are shown in Table 1). According to Hurley staging [10], the majority of patients were assessed as Hurley II (43%), followed by Hurley III (38.3%), and Hurley I (18.7%). In addition, the self-reported HS severity was recorded. On average, every patient had more than two body areas affected by the disease (2.5 ± 1.3 localizations) (Table 1).

Regarding treatment, most of the patients (318 patients, 92.9%) had already been given treatment, while the rest (24 patients) had been assessed but had not yet started any kind of therapy. Among those who had already been treated, 297 patients (86.8%) had been treated pharmacologically, while 193 individuals (56.4%) had undergone surgery.

### 2.2. Quality of Life

In order to evaluate the influence of the disease on the patients’ quality of life, all the participants were asked to complete the Hidradenitis Suppurativa Quality of Life-24 (HSQoL-24) questionnaire [8,9], a new Spanish HS-specific questionnaire which was also recently translated into and validated in English [9]. The instrument was a self-administered questionnaire consisting of 24 items that evaluate six life domains (psychosocial, economic, occupational, relationships, personal, and clinical) over a 4-week recall period [9]. Each item was scored on a five-point Likert scale. The total score was calculated by adding together the results from all the items, resulting in a maximum of 96 and a minimum of 0 points [9]. The higher the score, the bigger the impact of the disease on the patient’s quality of life. To convert the score to a percentage, it was necessary to multiply the scores by the following coefficients: total score, 1.0412; psychosocial, 2.08; economic, 25.0; employment, 12.5; relationships, 6.25; personal, 12.5; clinical, 8.33. Four cut-off values were introduced to classify the effect of HS on the QoL: 0–24, no effect; 25–32, small impairment; 32–43, moderate impairment; ≥44, serious impairment. Both the Spanish and English versions of the questionnaire showed very good internal consistency and reliability. The instrument presented high correlation coefficients with SKINDEX-29 and DLQI [9].

### 2.3. Statistical Analysis

A statistical analysis of the obtained results was performed via the use of the IBM SPSS Statistics v. 26 (SPSS INC., Chicago, IL, USA) software. All data were assessed for parametric or nonparametric distribution. The minimum, maximum, mean, and standard deviation were calculated. The quantitative variables were evaluated using the Mann–Whitney U test and the Spearman and Pearson correlations. For the qualitative data, the chi-squared test was used. Differences in the DLQI total score of patients with different HS severities according to the Hurley stages system were assessed using the Kruskal–Wallis one-way analysis of variance by ranks test. A two-sided *p*-value lower than 5% was considered to be significant.

## 3. Results

The perceived impairment of QoL due to HS in the studied group was considered to be serious, with a HSQoL-24 total score mean result of 58.3 ± 21.0 points. Similar results were observed for every life domain besides personal, for which the mean score was significantly lower (37.4 ± 25.1 points, indicating moderate QoL impairment) (Table 2). Women reported a significantly higher global QoL impairment total in comparison to men (61.6 ± 19.2 points vs. 51.1 ± 23.1 points, *p* < 0.001). Similarly, female patients scored significantly higher in the psychosocial, economic, relationships, and clinical domains. There was no statistically significant difference in QoL impairment between the sexes in the employment and personal domains (Table 2).

Differences in QoL impairment were also seen between different age groups. The global score of patients aged between 31 and 60 years was significantly higher than that of the rest of the groups (*p* = 0.002). Moreover, this statistically significant difference was also confirmed for the psychosocial, employment, and personal domains. Regarding the living situation, patients living with their family scored higher (indicating a higher level of QoL impairment) than other groups (62.1 ± 19.7 points vs. 48.9 ± 22.3 points vs. 26.6 ± 20.91, *p* < 0.001). This statistically significant difference was evident not only in the global score but also in the scores for the psychosocial, economic, employment, and clinical domains. Interestingly, the group whose QoL was least affected, according to the global score and the scores for every domain, were patients who lived alone (Table 3).

The level of education and the BMI did not have any influence on HS-associated QoL (detailed data are not shown). The analysis of the results according to employment situation revealed that the highest levels of QoL impairment were among the patients who were incapacitated by the disease and those who were currently unemployed. On the other hand, students presented the lowest levels of QoL impairment most frequently. 

The level of QoL impairment (based on the global score and the scores for each domain) correlated positively yet weakly with the number of affected body areas (for the global score, *r* = 0.285, *p* < 0.001). Similarly, the duration of the disease correlated positively with the HSQoL-24 total score (*r* = 0.173, *p* = 0.001), while the patients’ age at HS onset correlated negatively with the HSQoL-24 global score (*r* = −0.182, *p* = 0.001). The HS severity had a large influence on the level of QoL impairment; patients with a higher severity scored higher (69.0 ± 18.5 points) than those with a moderate (62.1 ± 17.6 points) or mild (46.0 ± 19.4 points) severity of the disease (*p* < 0.001). Surprisingly, these differences were not observed between patients at different Hurley severity stages. Regarding treatment, there was no difference in QoL impairment between patients receiving any kind of treatment and those who were not receiving any treatment at all. In contrast, patients who had undergone surgical treatment in the past tended to score significantly higher than those who had not (62.4 ± 19.6 points vs. 52.9 ± 21.6, *p* < 0.001).

## 4. Discussion

Hidradenitis suppurativa is a chronic, painful, debilitating, and recurrent inflammatory disorder affecting the pilosebaceous unit [11]. The incidence of HS is hard to estimate, as it fluctuates from 0.03% in the Japanese population [12] to 4% among German women [13]. The disease begins in early adulthood, usually after adolescence, with the peak incidence occurring in patients aged between 20 and 30 years [14]. It is characterized by the appearance of deep, inflammatory nodules; abscesses; fistulas; and scarring, predominantly in intertriginous areas such as the axillae, the perianal and inguinal areas, and skin folds [15]. The enormous effect that HS has on the QoL of patients, their families, and their partners due to the pain, itchiness, purulent discharge, and foul smell caused by the disease has been documented [16,17]. The disease is associated with a higher incidence of depression, suicidal thoughts, stigmatization, alexithymia, and unemployment [18,19,20,21].

Numerous dermatology-specific instruments have been implemented to measure the QoL impairment associated with HS. Among the most frequently used are Skindex [22], the Dermatology Life Quality Index (DLQI) [3], the EuroQol 5 Dimensions questionnaire (EQ-5D) [23], and the Short Form 36 questionnaire (SF-36) [24]. The largest study evaluating HS-associated QoL impairment, conducted on a group of 1795 patients, was recently published by our research group. [6]. The effect of HS on the patients’ QoL assessed via the DLQI was very large, with a mean DLQI score of 13.2 ± 8.1 points [6]. Similar yet slightly lower results were obtained in previous studies carried out by Matusiak et al. (12.7 ± 7.7 points) [16], Frings et al. (12 ± 7.0 points) [25], Jørgensen at al. (11.9 ± 7.6 points) [26], and Kourins et al. (11.43 ± 6.61 points) [27]. Nevertheless, it is important to underline that, due to the lack of HS-specific questions, none of the dermatology-specific QoL instruments adequately reflect the influence of HS on patients’ QoL. The first HS-specific questionnaire, named Hidradenitis Suppurativa Quality of Life (HiSQOL), was created by Thorlacius et al. [28] in 2019. The instrument was subsequently validated and translated into Polish [29,30]. Nevertheless, there is still no study confirming the utility of HiSQOL for implementation in clinical settings on a significant number of patients. At present, to the best of our knowledge, there is a total of six HS-specific QoL questionnaires available. Among them, according to the new consensus on QoL in HS patients presented by Chernyshov et al. [6], HIDRAdisk, HSIA, HiSQOL, and HSQoL-24 are the most valid, yet these instruments still lack real-life implementation.

Our study was the first to use the newly developed HS-specific questionnaire HSQoL-24 in a clinical setting among a large group of patients. The results of our study confirmed the burdensome character of the disease. The mean score of 58.3 ± 21.0 points indicated a serious impairment of patients’ QoL. Additionally, patients with a higher self-assessed level of HS severity tended to score significantly higher than those experiencing a mild severity of the disease. Furthermore, the number of affected areas had a significant influence on the perceived impairment of patients’ QoL. This result is in accordance with the results of studies performed using dermatology-specific instruments [6,16,25,26], yet direct comparison is impossible. Similarly, as in previously mentioned studies, significantly higher scores were noted in the female population. The difference in QoL impairment between the sexes was statistically significant for the total score as well as for almost every domain. This confirms the fact that women tend to suffer more from dermatological diseases, which has previously been confirmed in the cases of multiple dermatoses including vitiligo, psoriasis, and atopic dermatitis [31,32,33]. Interestingly, we found that patients who lived alone scored significantly lower than those living with their families. This may have been caused by the effect of the disease on the patients’ partners and family members [17]. According to the study by Włodarek et al. [17], HS has a moderate impact on the QoL of patients’ partners, and the effect correlates positively with the disease severity. Therefore, it seems that patients who live alone most probably do not feel the burden of their disease being imposed on their loved ones.

We are aware of the limitations of this study. The consideration of only self-assessed disease severity may have influenced the final results; however, the Hurley staging system was also employed. Moreover, we did not compare our results with the results of other instruments, as the aim of our study was to confirm the utility of the newly developed and validated HS-specific questionnaire in a real-life clinical setting. Moreover, future studies should be conducted in order to assess the questionnaire’s usefulness in measuring the treatment response.

## 5. Conclusions

In conclusion, this is the first study assessing the QoL impairment caused by HS using an HS-specific questionnaire. It shows that HSQoL-24 is a reliable tool for measuring the decrease in QoL in clinical settings. We believe that in the future, more studies regarding the effect of HS on patients’ quality of life should be performed with disease-specific, rather than dermatology-specific, questionnaires.

## Figures and Tables

**Table 1:** Patients’ characteristics.

Characteristics	Result
Sex, number of participants (%):	
Men	108 (31.6)
Women	234 (68.4)
Age, number of participants (%):	
30 years old or younger	90 (26.3)
31–60 years old	247 (72.2)
60 years old or older	5 (1.5)
Mean ± SD (years)	37.5 ± 10.7
Weight:	
Mean ± SD (kg).	82.0 ± 17.3
Height:	
Mean ± SD (cm).	167.4 ± 8.8
Body mass index (BMI):	
Mean ± SD (kg/m^2^).	29.3 ± 6.1
Living situation, number of participants (%):	
Alone	59 (17.3)
With family	189 (55.3)
With partner	94 (27.5)
Education level, number of participants (%):	
Primary	50 (14.6)
Secondary	142 (41.5)
College	150 (43.9)
Employment, number of participants (%):	
Student	48 (14.0)
Active	178 (52.0)
Retired	20 (5.8)
Unemployed	69 (20.2)
Incapacitated	27 (7.9)
Current HS severity, number of participants (%):	
Mild	129 (37.7)
Moderate	101 (29.5)
Severe	112 (32.7)
Hurley stage, number of participants (%):	
I	64 (18.7)
II	147 (43.0)
III	131 (38.3)
Duration of the disease, number of participants (%):	
Less than 5 years	73 (21.3)
Between 5 and 10 years	53 (15.5)
More than 10 years	216 (63.2)
Mean ± SD (years)	15.9 ± 10.7
Number of localizations:	
Mean ± SD	2.5 ± 1.3
HSQoL-24 result:	
Mean ± SD (points).	58.3 ± 21.0
HSQoL-24 domains, mean ± SD (points):	
Psychosocial	58.9 ± 21.6
Economic	50.8 ± 36.2
Occupational	63.0 ± 31.3
Relationships	66.0 ± 27.8
Personal	37.4 ± 25.1
Clinical	62.9 ± 25.7

SD—standard deviation, BMI—body mass index, HSQoL-24—Hidradenitis suppurativa Quality of life 24.

**Table 2:** Comparison of HSQoL-24 scores between genders.

Domain, Mean ± SD (Points)	Men, *n* = 108	Women, *n* = 234	*p*
HRSQoL-24 Global	51.1 ± 23.1	61.6 ± 19.2	<0.001
HRSQoL-24 Psychosocial	51.3 ± 23.6	62.3 ± 19.7	<0.001
HRSQoL-24 Economic	39.3 ± 35.5	56.0 ± 35.3	<0.001
HRSQoL-24 Occupational	58.9 ± 33.3	64.9 ± 30.2	0.102
HRSQoL-24 Relationships	57.0 ± 27.7	70.2 ± 26.8	<0.001
HRSQoL-24 Personal	35.0 ± 27.3	38.5 ± 24.0	0.253
HRSQoL-24 Clinical	56.6 ± 25.8	65.8 ± 25.1	0.002

HSQoL-24—Hidradenitis Suppurativa Quality of Life-24; *n*—number of participants; SD—standard deviation.

**Table 3:** HSQoL-24 scores depending on living situation.

Domain, Mean ± SD (Points)	Alone, *n* = 59	With Family, *n* = 189	With Partner, *n* = 94	*p*(ANOVA)
HRSQoL-24 Global	48.9 ± 22.3	62.1 ± 19.7	56.6 ± 20.91	<0.001
HRSQoL-24 Psychosocial	49.6 ± 21.9	63.0 ± 20.0	56.3 ± 22.6	<0.001
HRSQoL-24 Economic	42.8 ± 37.7	57.8 ± 33.9	41.7 ± 36.9	<0.001
HRSQoL-24 Occupational	48.1 ± 31.7	66.9 ± 29.9	64.5 ± 31.3	<0.001
HRSQoL-24 Relationships	58.8 ± 38.7	69.0 ± 24.4	64.4 ± 25.3	0.04
HRSQoL-24 Personal	30.4 ± 23.7	39.9 ± 25.1	36.7 ± 25.3	0.039
HRSQoL-24 Clinical	52.5 ± 28.4	65.2 ± 23.7	64.8 ± 26.2	0.003

HSQoL-24—Hidradenitis Suppurativa Quality of Life-24; *n*—number of participants; SD—standard deviation.

## Data Availability

Data sharing not applicable due to ethics policy.
